# Mechanisms regulating cerebral hypoperfusion in cerebral autosomal dominant arteriopathy with subcortical infarcts and leukoencephalopathy

**DOI:** 10.7555/JBR.36.20220208

**Published:** 2022-08-28

**Authors:** Xi Yan, Junkui Shang, Runrun Wang, Fengyu Wang, Jiewen Zhang

**Affiliations:** Department of Neurology, Henan Provincial People's Hospital, Zhengzhou University People's Hospital, Henan University People's Hospital, Zhengzhou, Henan 450003, China

**Keywords:** cerebral hypoperfusion, CADASIL, Notch3, mural cell, astrocyte

## Abstract

Cerebral small vessel disease (CSVD) is a leading cause of stroke and dementia. As the most common type of inherited CSVD, cerebral autosomal dominant arteriopathy with subcortical infarcts and leukoencephalopathy (CADASIL) is characterized by the *NOTCH3* gene mutation which leads to Notch3 ectodomain deposition and extracellular matrix aggregation around the small vessels. It further causes smooth muscle cell degeneration and small vessel arteriopathy in the central nervous system. Compromised cerebral blood flow occurs in the early stage of CADASIL and is associated with white matter hyperintensity, the typical neuroimaging pathology of CADASIL. This suggests that cerebral hypoperfusion may play an important role in the pathogenesis of CADASIL. However, the mechanistic linkage between *NOTCH3* mutation and cerebral hypoperfusion remains unknown. Therefore, in this mini-review, it examines the cellular and molecular mechanisms contributing to cerebral hypoperfusion in CADASIL.

Cerebral autosomal dominant arteriopathy with subcortical infarcts and leukoencephalopathy (CADASIL) is a common pure form of subcortical vascular dementia, which is caused by *NOTCH3* gene mutation. Granular osmiophilic material (GOM) deposition in the small arteries is the classical pathology of CADASIL^[1]^. White matter hyperintensity (WMH), especially that in the anterior temporal lobes and external capsule, has been identified as a typical neuroimaging feature of CADASIL^[2]^. Currently, many mechanisms are reported to be linked to WMH, including cerebral hypoperfusion, impaired cerebral vascular reactivity, and blood-brain barrier impairment. Among them, cerebral hypoperfusion is an early feature of the disease and its relationship with WMH has been clearly investigated by many groups^[3]^. Collectively, these studies provide strong evidence demonstrating that cerebral hypoperfusion is an early event and may play a key role in the CADASIL pathophysiological cascade^[4]^. In this review, we summarize the possible pathophysiology of hypoperfusion in CADASIL.

Although there is sufficient evidence confirmed the existence of cerebral hemodynamic abnormalities and cerebral arteriolar dysfunction in CADASIL patients, how *NOTCH3* gene mutation causes arteriolar dysfunction has not been clearly studied. The mature Notch3 receptor consists of the 210-kDa Notch3 extracellular domain (Notch3^ECD^) and the 97-kDa Notch3 transmembrane intracellular domain (Notch3^TICD^). Signal activation of Notch3 requires the Notch3 receptor to undergo a series of proteolytic cleavages for division into Notch3^ECD^ and Notch3^TICD[5]^. Notch3 protein is mainly expressed in mural cells, including vascular smooth muscle cells (VSMCs) and pericytes^[6]^. Therefore, it can be speculated that abnormal vascular mural cells may be an important reason for the dysfunction of arterioles in CADASIL.

Accumulation of Notch3^ECD^ is one of the earliest detectable pathological features in CADASIL patients and mouse models^[4]^, suggesting that Notch3^ECD^ accumulation is an early event in CADASIL. *NOTCH3* mutations lead to an odd number of cysteine residues in Notch3^ECD^, consequently resulting in abnormal degradation of Notch3^ECD^ on the surface of mural cells. Joutel A. is the first to put forward the Notch3^ECD^ cascade hypothesis for CADASIL: early Notch3^ECD^ deposition abnormally recruits the other extracellular matrix (ECM) proteins, thus leading to the accumulation and formation of the so-called GOM, which can be observed under the transmission electron microscope^[7]^. Notch3^ECD^ along with other ECM proteins causes dysfunction in the small vessels of the brain. This hypothesis provides an alternate understanding of the pathogenesis of CADASIL. Postmortem studies have found a series of ECM proteins that are components of GOM, including clusterin, tissue inhibitor of metalloproteinases 3 (TIMP3), vitronectin, latency in TGF-β binding protein 1 (LTBP-1), high-temperature requirement protein A1 (HTRA1), and other ECM proteins^[8]^. Abnormal deposition of these proteins can affect the normal structure and function of arterioles. TIMP3 is a tissue inhibitor of matrix metalloproteinase, which helps maintain tissue microenvironment homeostasis^[9]^. Studies have shown that the increase of TIMP3 is associated with abnormal small artery function and decreased cerebral blood flow (CBF) in mouse models^[10]^. Abnormal TIMP3 accumulation inactivates the metalloprotease ADAM17, which results in the inactivation of HB-EGF-ErbB1/ErbB4 signaling and endocytic dysfunction of the voltage-dependent potassium channels (Kv). Increased Kv channel activity in the VSMCs membrane leads to compromised cerebral arterial tone and CBF responses, which can be restored by exogenous addition of ADAM17 or HB-EGF^[11]^. Besides, an autophagy-lysosomal defect was detected in human CADASIL VSMCs^R133C[12]^, aggravating the accumulation of intracellular and extracellular proteins. Abnormal aggregation and deposition of proteins can further lead to endoplasmic reticulum oxidative stress, apoptosis and cell dysfunction^[13]^.

Notch3 signaling plays an important role in arterial maturation, maintenance, and function^[14]^. Given its critical role in vascular integrity, dysregulation of Notch3 signaling may be associated with cerebrovascular dysfunction in individuals with *NOTCH3* gene mutations. Severe cerebrovascular abnormalities have been detected in *NOTCH3* gene knockout mice, including VSMC loss, blood-brain barrier leakage, and arterial myogenic tone changes^[14]^. Interestingly, cerebrovascular abnormalities reported in *NOTCH3* knockout mice were consistent with those observed in CADASIL mutant mice (TgNotch3^C455R^)^[15]^. However, except for mutations in the binding region of Notch3 receptor, the Notch3 pathway activity is not significantly affected^[16–17]^. Besides, increased Notch3 activity and the impairment of maximal vasodilator capacity were found in CADASIL mutant mice (TgNotch3^R169C^), which can be prevented by conditionally reducing Notch3 activity and mimicked by conditionally activating Notch3 in VSMCs^[18]^. Therefore, different *NOTCH3* mutations may have different effects on Notch3 signaling, and contribute to reductions in CBF through different mechanisms.

In addition to mural cells, Notch3 receptor is also expressed in astrocytes. Being a part of the cellular composition of the neurovascular unit (NVU), astrocytes contribute to the regulation of small vessel tone and CBF responses^[19]^. In 2010, Brennan-Krohn T *et al* reported that astrocytic-associated molecular abnormalities are restricted to the regions of WMH and contribute to vascular degeneration in brain tissues in patients with CADASIL^[20]^. Furthermore, in 2018, Hase Y *et al* demonstrated the activation of normal-appearing astrocytes and their transformation into clasmatodendritic astrocytes in the deep white matter in CADASIL^[21]^ (***Fig. 1***). Immunohistochemical analyses have shown that clasmatodendritic astrocytes are abundant in the anterior temporal pole and that aquaporin 4 (AQP4) is displaced in these astrocytes^[21]^, indicating that the gliovascular unit of the deep white matter is severely impaired in CADASIL (***Fig. 1***). Next, we tried to understand the pathology of neurovascular dysfunction and WMH in light of astrocytopathy in CADASIL.

**Figure 1:**
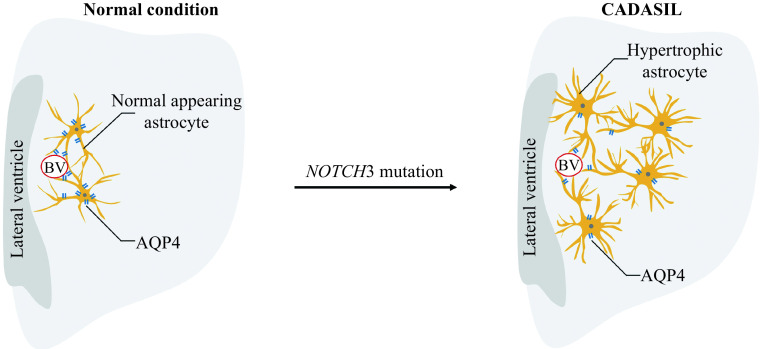
Astrocytopathy in the deep WM of CADASIL.

Astrocyte endfeet show close apposition around VSMCs and pericytes. Increased intracellular Ca^2+^ levels in astrocyte endfeet result in arteriolar vasodilation^[22]^. A study has reported the astrocyte-regulated vascular tone in VSMC through the phospholipase A2-arachidonic acid pathway^[23]^ (***Fig. 2***). Whether astrocytopathy-mediated VSMC dysfunction is the cause of CBF reduction in CADASIL remains unknown.

**Figure 2:**
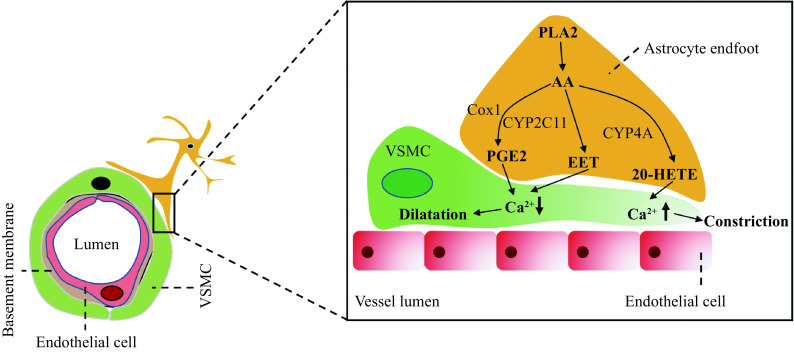
Summary of signaling pathways involved in astrocyte-mediated regulation of the VSMC tone.

Astrocyte endfeet also form a unique network of interconnected perivascular tunnels called the glymphatic system^[24]^ (***Fig. 3***). The function of the perivascular glymphatic pathway is primarily dependent on AQP4 water channels, which are highly expressed in the astrocyte endfeet. The glymphatic system not only facilitates the elimination of excess fluid and interstitial waste in the brain parenchyma but also plays an important role in the fluid exchange between cerebrospinal fluid (CSF) and brain parenchyma interstitial fluid (ISF). Waste clearance and fluid exchange are critical for brain homeostasis maintenance^[24]^. WMH represents local fluid accumulation in the white matter. Decreased glymphatic activity probably results in waste and fluid accumulation and further initiates the loss of mature oligodendrocytes and WMH in patients with CADASIL^[25]^ (***Fig. 3***). WMH is not only the hallmark of CADASIL, but also the characteristic pathological change in other small-vessel diseases (SVDs). The stroke-prone spontaneously hypertensive rat (SHRSP) is a rodent model of human sporadic SVD. Studies show that total CSF flux is significantly reduced in the SHRSP rats than in controls. The glymphatic flux is also significantly reduced in SHRSP rats than in controls. Altered perivascular AQP4 expression is also observed in SHRSP^[26]^. Diabetes is a cardiovascular risk factor for SVD. Dysfunctional CSF flow dynamics and glymphatic transport occur in the streptozotocin-induced type 2 diabetes rat model^[27]^.

**Figure 3:**
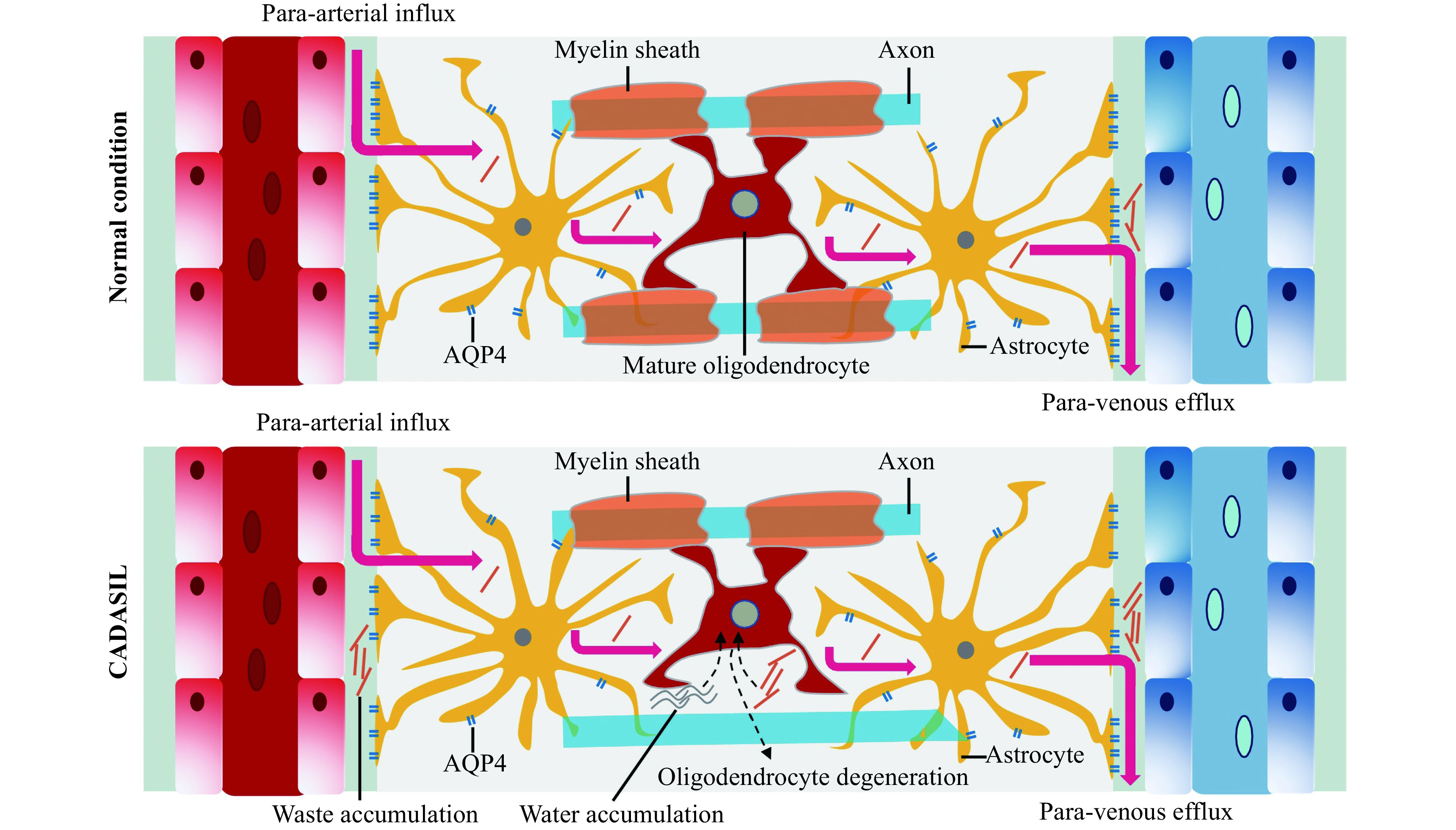
Model of glymphatic transport in healthy condition population and CADASIL patients.

Recent studies have demonstrated that CBF hemodynamic deficit occurs in the early stage of CADASIL and plays an important role in brain parenchymal injury formation. As the component of NVU, endothelial cell, VSMC, pericyte, astrocyte and ECM all participate in CBF regulation. We reviewed the possible pathophysiology of hypoperfusion in CADASIL from the aspects of mural cells and glial cells. *NOTCH3* gene mutations cause abnormal Notch3 deposition and signaling, leading to mural cell dysfunction and degeneration. However, whether severe astrocytopathy is the main cause of vascular degeneration or it is secondary to VSMC- and pericyte-mediated vascular pathology in the deep white matter of CADASIL remains to be elucidated. Much more work is needed to elucidate the relationship between *NOTCH3* mutations and decreased CBF in order to seek therapeutic targets and clinical translations.
